# 1751. The Conundrum of Neonatal Herpes Simplex Virus (HSV) Disease: Co-infections do Happen!

**DOI:** 10.1093/ofid/ofad500.1582

**Published:** 2023-11-27

**Authors:** Asuncion Mejias, Jeanette Taveras, Alvaro A Dendi, Traci Pifer, Rachelle Crisan, Pablo J Sanchez

**Affiliations:** St Jude Children's Researh Hospital, Memphis, Tennessee; Nationwide Children's Hospital, Columbus, Ohio; Facultad de Medicina, Universidad de la Republica, Montevideo, Montevideo, Uruguay; Nationwide Children's Hospital, Columbus, Ohio; Nationwide Children's Hospital, Columbus, Ohio; Nationwide Children's Hospital - The Ohio State University, Columbus, OH

## Abstract

**Background:**

Traditional dogma states that identification of one pathogen in neonates undergoing sepsis evaluation excludes other infections like HSV. At Nationwide Children’s Hospital Columbus, OH, all neonates with suspected sepsis are evaluated for bacterial and viral infections including HSV disease. The frequency of coinfection with HSV when another pathogen is identified in an ill-appearing neonate is not fully known, yet timely treatment is paramount to lessen HSV morbidity and mortality.

**Table 1.**
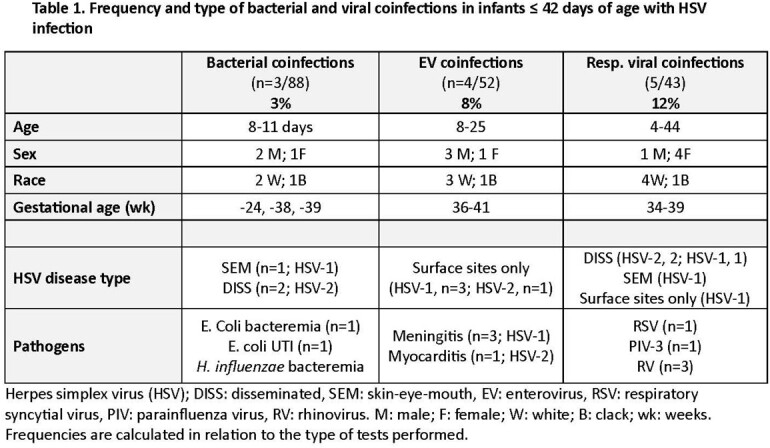
Frequency and type of bacterial and viral coinfections in infants ≤ 42 days of age with HSV infection

**Methods:**

From 1/2007-12/2022, infants ≤ 42 days old with a positive HSV PCR from blood, cerebrospinal fluid (CSF), or mucosal sites were identified by ICD-9/10 codes (before 2012) and subsequently by prospective surveillance. Information regarding bacterial cultures and/or viral PCRs, clinical and outcome data were reviewed. Infants coinfected with HSV and another viral or bacterial pathogen were identified.

**Results:**

We identified 88 infants who tested positive for HSV by PCR of a body site and had bacterial and other viral studies performed. The rate of coinfections was 14% (12/88; **Table 1**). Bacterial cultures were positive in 3% (3/88) of infants: a full-term 11-day old with *E. coli* UTI and HSV-1 skin-eye-mouth (SEM) disease; a full term 11-day old with *H. influenzae* bacteremia and HSV-2 disseminated (DISS) disease, and a 24 wks’ gestation infant with *E. coli* sepsis who died at 8 days of age with HSV-2 DISS disease. Viral co-detection included positive enterovirus (EV) PCR in 8% (4/52) of infants (3, CSF; 1, blood/mucosal sites). All four had HSV DNA detected from mucosal sites only: 3 HSV-1 and EV PCR + in CSF on days 6-25 of age and an infant with EV myocarditis [EV blood PCR +] at 8 days of age and HSV-2. A respiratory virus was identified in 12% (5/43) of infants: 3 with DISS disease (2, HSV-2; 1, HSV-1) born at 35, 37, and 34-wks’ and had concomitant rhinovirus (RV; died), RSV, and parainfluenza-3 infection at 4, 30, and 31 days old, respectively. The remaining 2 infants had RV infection and HSV-1 SEM (44 days old) and HSV-1 mucosal site infection (11 days old).

**Conclusion:**

14% of neonates with HSV infection had concomitant bacterial, respiratory viral, or enteroviral disease. The identification of a pathogen besides HSV in neonates with suspected sepsis does not rule out HSV disease.

**Disclosures:**

**Asuncion Mejias, MD, PhD, MsCS**, Astra-Zeneca: Advisor/Consultant|Merck: Grant/Research Support|Pfizer: Advisor/Consultant|Sanofi-Pasteur: Advisor/Consultant

